# Five years comparation of efficacy and safety after ICL-V4c implantation for high and super high myopia correction

**DOI:** 10.1080/07853890.2024.2448282

**Published:** 2024-12-30

**Authors:** Qi Wan, Li Chen, Peiyuan He, Ran Wei, Ke Ma, Hongbo Yin, Jing Tang, Ying-ping Deng

**Affiliations:** a Department of Ophthalmology, West China Hospital of Sichuan University, Chengdu City, China; b Department of Health Management & Institute of Health Management, Sichuan Provincial People’s Hospital, University of Electronic Science and Technology of China, Chengdu City, China

**Keywords:** High myopia, super-high myopia, posterior chamber phakic, implantation, ICL-V4c

## Abstract

**Objectives:**

The objective of the investigation is to examine the long term efficacy, safety, and predictability of ICL-V4c implantation for high and super-high myopic patients in order to provide reliable guidance for the selection of refractive surgical procedures.

**Methods:**

We reviewed 125 eyes from 64 patients who implanted ICL-V4c at the Refractive Surgery Center of West China Hospital in Chengdu, China, between May 2015 and January 2017. These eyes were divided into two groups based on their preoperative spherical equivalent (SE) degree: high myopia (≥ −10D) and super-high myopia groups (< −10D). We followed up with the patients over 5 years and evaluated several parameters, including uncorrected visual acuity (UDVA), corrected visual acuity (CDVA), axial length (AL), refractive error, endothelial cell density (ECD), intraocular pressure (IOP), white-to-white distance (WTW), and vault.

**Results:**

The efficacy indices of ICL-V4c implantation in high and super-high myopia groups were 0.91 ± 0.23 and 0.80 ± 0.25, respectively at 5 years after operation. Compared to high myopia group, the efficacy index of super-high myopia was obviously decreased (*p* = 0.020) and the △AL of super-high myopia was significantly increased (*p* = 0.001). The mean safety indices were 1.10 ± 0.15 and 1.10 ± 0.21 respectively in high and super-high myopia groups (*p* = 0.850). At the 5-year mark, 11.67% vs 20.00% (High vs Super-high) of eyes were within ±0.50 D (Spherical Equivalent), and 75.00% vs 70.77% (High vs Super-high) of eyes were within ±2.00 D. No significant difference of ECD was found in the high (2823.45 ± 274.75 cells/mm^2^) and super-high myopia (2856.71 ± 323.53cells/mm^2^) at the visit of 5 years. Compared to baseline, we observed a significant increase in IOP at the 1-week follow-up, which decreased significantly at the one-month visit. Furthermore, there was a significant difference of vault between the high and super-high groups at 1-month (*p* = 0.042) and 5-year (*p* = 0.002) after surgery.

**Conclusions:**

ICL-V4c implantation is effective, safe, and stable for correcting high and super-high myopia. However, ophthalmologists need to be aware of the potential for greater myopia regression in super-high myopic patients, as well as the increase in axial length and associated fundus complications.

## 1. Background

Based on the World Health Organization’s study report, almost 600 million individuals have myopia, with around 5% having high or ultra-high myopia. According to the incidence rate, the number of myopia patients worldwide would surpass 5 billion by 2050, with the number of high myopic and ultra-high myopic patients increasing to 938 million [1,2]. Patients with high and ultra-high myopia require particular treatment since they lack confidence and have few professional options [3–5]. Therefore, surgery can not only correct refractive errors, but also bring significant changes in personality, career, and lifestyle. However, for patients with high myopia, the corneal laser refractive surgery approach is not applicable for some patients at the current stage, as corneal thickness is too thin and myopic degree is too high [6,7].

Implantable Collamer Lens (ICL) surgery can solve these problems by implanting the ICL lens into the ciliary sulcus. However, early clinical verification of refractive lens surgery showed that some patients developed complications such as cataracts, loss of corneal endothelial cells, and secondary glaucoma [8–10]. These complications were caused by factors such as iridotomy during surgery, contact between the implanted lens and the anterior capsule, and friction with the corneal endothelial cell layer or obstruction of aqueous humor outflow. To solve these complications, a central-hole ICL was developed with a hole diameter of 360 μm, which increases the flow rate of aqueous humor in a more natural state and facilitates communication between the anterior and posterior chambers [11–13]. This lens is named ICL-V4c and has a special arched design that reduces the chance of contact with the crystalline lens capsule, thereby reducing cataract formation. ICL-V4c has been introduced to China for less than 10 years, and its use time is not long. A comprehensive review of the literature reveals a consistent pattern of positive outcomes following ICL-V4c implantation. Several studies have reported significant improvements in visual acuity, refractive error, and patient satisfaction in high myopia patients. The ICL-V4c has demonstrated excellent refractive stability over long-term follow-up periods, with a low rate of regression or loss of efficacy [8,14–19]. Doctors and patients, on the other hand, have concerns about its long term safety, efficacy, and stability.

There are presently no long-term comparison reports on the use of ICL-V4c for treating super-high myopic patients to our knowledge. Super-high myopic patients often have complications such as posterior scleral protrusion, elongation of the axial length, and myopic maculopathy. As a consequence, this study observed the effectiveness, accuracy, safety, and stability of ICL-V4c implantation in correcting high myopic and super-high myopic patients for 5-year postoperatively to evaluate objectively the long term refractive and visual results of ICL-V4c in correcting super-high myopic patients.

## 2. Methods

### 2.1. Patients

A total of 212 patients had the implantation surgery of ICL-V4c at Refractive Surgery Center of West China Hospital in Chengdu, China, between May 2015 and January 2017. Among them, 64 patients with 125 eyes were included in the final analysis, as they were able to return to the hospital for follow-up examination *via* phone recall. These patients were classified into two groups based on their preoperative spherical equivalent (SE) degree: those with a preoperative SE greater than −10D were classified as high myopia group, while those with a preoperative SE degree less than or equal to −10D were classified as super-high myopia groups [20,21]. The high myopia group included 60 eyes (SE range of −3D to −9.75D, with a mean of −8.89 D), of which 27 eyes had astigmatism (cylindrical range of −0.5D to −6D), while the super-high myopia group included 65 eyes (SE range of −10D to −20.5D, with a mean of −13.59 D), of which 40 eyes had astigmatism (cylindrical range of −0.5D to −5.5D).

### 2.2. Inclusion and exclusion criteria

Inclusion criteria for this study were: (1) between the ages of 18–45 years; (2) possessing a stable refraction with an annual increase of no more than 0.50 diopter for at least two consecutive years; (3) and avoiding contact lens use for a minimum of 2 weeks or rigid gas permeable contact lenses for at least 4 weeks prior to surgery; (4) All patients provided written consent forms. and adhered to principles established by the Helsinki Declaration. The Ethics Committee of West China Hospital approved this research.

The exclusion criteria including: (1) patients who are pregnant or breastfeeding; (2) patients with a history of ocular or systemic diseases, such as progressive corneal degeneration, ocular surgery, glaucoma, cataract, diabetic retinopathy or uveitis, were excluded from this study; (3) individuals with preoperative anterior chamber depth less than 2.8 mm or preoperative endothelial cell density less than 2000 cells/mm^2^ were also excluded from this study.

### 2.3. Pre‑ and postoperative measurements

This study collected both pre- and post-operative ophthalmologic measurements. The follow-up period for this study was 5 years, during which uncorrected distance visual acuity (UDVA, logMAR) and corrected distance visual acuity (CDVA, logMAR) were recorded. Additionally, the study evaluated other parameters such as slit-lamp examination, fundus examination, manifest refractive error, intraocular pressure (IOP, the Canon Full Auto Tonometer, Tokyo, Japan), axial length (AL, IOL-master 500, Carl Zeiss, Germany), and endothelial cell density (ECD, Topcon SP-3000P, Tokyo, Japan). The Pentacam HR (Oculus Optikgerate, Wetzlar, Germany) was also used to assess central corneal thickness (CCT), white-to-white distance (WTW), keratometry steep (Ks), keratometry flat (Kf), pupil diameter (PD), and anterior chamber depth (ACD) without the use of pupil dilation. The refractive surgery impact on quality of life questionnaire (QIRC) consists of four main modules were also administered: (1) Eye Sensation, Symptom and Visual Function; (2) Physical Fitness; (3) Social Activity; (4) Psychological and Mental Health – with a total of 20 questions [22]. Under the guidance of the data collector, all patients were asked to select their current eye and psychological/mental conditions after ICL-V4c implantation surgery. Each item is divided into five levels (none, mild, moderate, severe, extreme). The final score is calculated to observe patient satisfaction with the surgery.

### 2.4. Surgical procedure

The manufacturer calculated the power for the ICL-V4c using a modified vertex formula based on the preoperative refractive characteristics supplied. The WTW horizontal corneal diameter and anterior chamber depth, as well as a reference to sulcus-to-sulcus assessed by ultrasonogram biomicroscopy (UBM), were used to calculate the size of the implanted ICL-V4c.

All surgeries were carried out by an experienced surgeon (YPD) who followed standardized methods based on earlier research. During surgery, a 2.7-mm temporal corneal incision was performed at the temporal corneoscleral limbus. Following the injection of Healon (1% Sodium hyaluronate, Bausch & Lomb, China) to sustain the anterior chamber depth, the ICL-V4c was introduced into the anterior chamber with an injector cartridge. A viscoelastic agent was put over the ICL in certain cases, and an ICL positioning tool was utilized to sweep the four haptics of the ICL beneath the iris. Ultimately, the sodium hyaluronate was irrigated with balanced salt solution, and the incision was hydrated and examined for water tightness with a balanced salt solution. Patients were given steroid eye drops four times a day for 1 week, antibiotic eye drops, and artificial tears four times a day for 2 weeks after surgery.

### 2.5. Statistical analysis

SPSS software (version 26.0) and R language (version 4.1.2) were used for data analysis and visualization. The efficacy is defined as post-UDVA/pre-CDVA; The safety is defined as post-CDVA/pre-CDVA [8,18,23,24]. The normally distributed continuous variables were described using the mean and standard deviation (*SD*), while paired t-tests were applied for pre‑ and postoperative comparisons within the group and independent sample t-tests were used for between-group comparisons. Categorical data were presented as frequencies and percentages, with between-group comparisons analyzed using chi-square (*χ*^2^) tests. In instances where the chi-square test conditions were not met, Fisher’s exact probability test was used. Differences were considered statistically significant if *p* < 0.05. The ggplot2 package in R language was utilized for all graphical representations.

## 3. Results

### 3.1. Baseline characteristics

A total of 64 myopia patients, consisting of 125 eyes, who had undergone ICL-V4c surgeries and followed for at least 5 years, were included in this retrospective study. Of these eyes, 40 eyes were treated with toric ICL-V4c and 85 eyes with ICL-V4c. The average UDVA was 1.49 ± 0.21 for the high myopia group and 3.64 ± 1.34 for the super-high group, while the mean CDVA was 0.00D for high myopia patients and 0.03D for super-high patients. The average age was 32.16 ± 6.70 years for the high myopia and 31.03 ± 6.65 years for the super-high myopia. The average preoperative spherical equivalent (SE) was −8.89 ± 1.94 D for high myopia patients and −13.59 ± 2.01 D for super-high myopia patients. The mean preoperative central corneal thickness (CCT) was 485.28 μm for high myopia patients and 518.62 μm for super-high patients. The p-values between the two groups for preoperative UCVA, CCT, and preoperative CDVA, SE were all less than 0.05. The baseline characteristics of data for the two groups are listed in Table 1

**Table 1.** Preoperative patient demographics (mean ± standard deviation).

Characteristics	high myopia	Super-high myopia	
Eyes	*N = 60*	*N = 65*	*p*
Age	32.16 (6.70)	31.03 (6.65)	0.501
UDVA (logMAR)	1.49 (0.21)	3.64(1.34)	<0.001
CDVA (logMAR)	0.00 (0.04)	0.03 (0.08)	0.006
Refraction cylinder (D)	−0.90 (1.35)	−0.56 (5.26)	0.617
Spherical equivalent (D)	−8.89 (1.94)	−13.59 (2.01)	<0.001
K-flat (D)	43.63 (1.53)	43.04 (1.61)	0.039
K-steep (D)	45.02 (1.65)	44.18 (1.64)	0.005
CCT (mm)	485.28 (29.34)	518.62 (68.09)	<0.001
Corneal diameter (mm)	11.60 (0.37)	11.65 (0.37)	0.463
Pupil diameter (mm)	3.86 (0.58)	3.97 (0.72)	0.344
ACD (mm)	3.28 (0.23)	3.25 (0.26)	0.605
ICL size (mm)	12.70 (0.37)	12.78 (0.36)	0.282

UDVA: uncorrected distance visual acuity; CDVA: corrected distance visual acuity; CCT: central corneal thickness; ACD: anterior chamber depth.

### 3.2. Efficacy (post-UDVA/pre-CDVA) and safety (post-CDVA/pre-CDVA/)

In high myopia group, we observed that the mean (±*SD*) efficacy indices were 1.18 (0.23), 1.19 (0.25), 1.09 (0.26), 1.08 (0.25), 1.13 (0.24) and 0.91 (0.23) at the postoperative 1-week, 1-month, 3-month, 6-month, 1-year and 5-year, respectively. The mean (±*SD*) efficacy indices in super-high myopia patients were 1.12 (0.21), 1.16 (0.23), 1.16 (0.22), 1.02 (0.22), 1.01 (0.19) and 0.80 (0.25) at the visit of 1-week, 1-month, 3-month, 6-month, 1-year and 5-year, respectively (Table 2). Compared to the high myopia group, the efficacy index of 5 years in super-high myopia patients has a significant decrease. The final follow-up found that the average safety indices were 1.10 (0.15) in high myopia group and 1.10 (0.21) in super-high myopia patients.

**Table 2.** The efficacy and safety of ICL-V4c implantation during the 5 years follow-up.

	ALL	High	Super High	*p*
Efficacy index				
1 week	1.15 (0.22)	1.18 (0.23)	1.12 (0.21)	0.139
1 month	1.18 (0.24)	1.19 (0.25)	1.16 (0.23)	0.549
3 months	1.14 (0.24)	1.09 (0.26)	1.16 (0.22)	0.292
6 months	1.05 (0.23)	1.08 (0.25)	1.02 (0.22)	0.390
1 year	1.06 (0.22)	1.13 (0.24)	1.01 (0.19)	0.102
5 years	0.85 (0.24)	0.91 (0.23)	0.80 (0.25)	0.020
Safety index				
5 years	1.10 (0.18)	1.10 (0.15)	1.10 (0.21)	0.850

**Figure 1.**
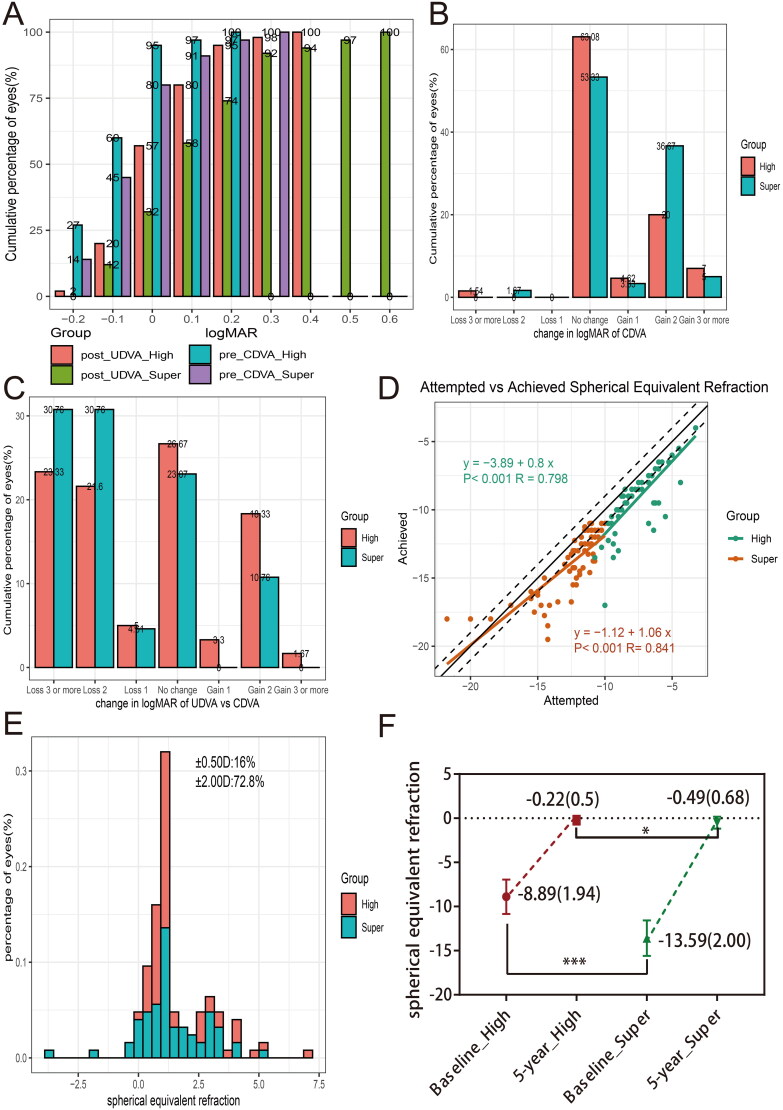
Clinical outcomes of high and super-high myopia groups at last follow-up after implantation of ICL-V4c. (A) Postoperative UDVA versus preoperative CDVA in high and super-high myopia groups. (B) Change in CDVA. (C) Difference between postoperative UDVA and preoperative CDVA for high and super-high myopia groups. (D) Attempted spherical equivalent refraction change versus the achieved spherical equivalent refraction change. (E) Distribution of postoperative spherical equivalent refraction accuracy. (F) Stability of spherical equivalent refraction at 5-year follow-up. CDVA: corrected distance visual acuity; UDVA: uncorrected distance visual acuity.

Our observational study found that 80% of the eyes assessed had achieved a postoperative UDVA equal to or better than 0.1 in high myopia group, while only 58% of the eyes in super-high myopia patients had achieved a postoperative UDVA ≥ 0.1. Additionally, all eyes included in the study demonstrated a postoperative UDVA equal to or better than 0.6 (Figure 1(A)). Notably, almost all of eyes exhibited the same or improved CDVA compared to their preoperative CDVA, with two eyes experiencing a decrease in CDVA (Figure 1(B)). A total of 49.97% of eyes in high myopia group demonstrated the same or improvement of at least one line, while 33.83% of eyes had same or gain at least one line in super-high group. 66.17% of super-high myopic eyes showed a loss of at least one line (Figure 1(C)).

### 3.3. Predictability and stability

Figure 1(D) presents a scatter plot illustrating the relationship between achieved and attempted spherical equivalent (SE) in high and super-high groups. The fitted curve of attempted and achieved SE in high myopia was *Y* = −3.89 + 0.8*X*. The fitted curve in super-high myopia was *Y* = −1.12 + 1.06*X*. At the final follow-up, it was observed that 11.67% vs 20.00% (High vs Super-high) of eyes were within ±0.50 D, and 75.00% vs 70.77% (High vs Super-high) of eyes were within ±2.00 D when comparing preoperative SE with the target refractive power (as displayed in Figure 1(E)). There was on differential distribution of predictability between high and super-high groups (Table 3). Moreover, Figure 1(F) indicates that the average dioptre of SE at the final follow-up was −0.22 ± 0.5 in high myopia group. While the average dioptre of SE was −0.49 ± 0.68 in super-high myopia group at the final follow-up.

**Table 3.** The accuracy of postoperative spherical equivalent refraction.

	[ALL]	High	Super high	
	*N = 125*	*N = 60*	*N = 65*	*p*-Value
Within ±0.50 D:				0.446
No	105 (84.00%)	53 (88.33%)	52 (80.00%)	
Yes	20 (16.00%)	7 (11.67%)	13 (20.00%)	
Within ±0.20 D:				0.868
No	34 (27.20%)	15 (25.00%)	19 (29.23%)	
Yes	91 (72.80%)	45 (75.00%)	46 (70.77%)	

### 3.4. Keratometry and axial length (AL)

The pre-operative mean *Kf* of high myopia group was 43.63D and 43.68D at 5-year postoperatively. The mean *Kf* of super-high myopia group was 43.04D at baseline and 42.91D at 5-year postoperatively. The preoperative and 5-year postoperative mean Ks were 45.02D and 45.14D in high myopia group. Meanwhile, in super-high myopia group, the preoperative and 5-year postoperative mean Ks were 44.18D and 44.12D, respectively. There was a significant difference between high and super-high myopia for Kf and Ks, while on significant difference was observed between pre-operation and 5-year post-operation. Besides, we surveyed that the AL of high myopia group increased from 26.14 mm preoperatively to 26.49 mm at 5-year postoperatively. The AL of super-high myopia group increased from 28.15 mm preoperatively to 28.48 mm at 5-year postoperatively. Compared to the △AL of high myopia, super-high myopia was significantly increased in AL (Table 4). We furtherly evaluated the correlation between the increase in axial length and preoperative parameters in myopic patients. We found that only the preoperative spherical equivalent was negatively correlated with the increase in axial length, consistent with previous research (Table 5).

**Table 4.** The pre- and post-operative keratometry and axial length (mean ± standard deviation).

	ALL	High	Super High	
Parameters	*N = 125*	*N = 60*	*N = 65*	*p*
Pre-Kf	43.32 (1.60)	43.63 (1.53)	43.04 (1.61)	0.039
Post-Kf (5-year)	43.28 (1.57)	43.68 (1.56)	42.91 (1.50)	0.006
Pre-Ks	44.58 (1.69)	45.02 (1.65)	44.18 (1.64)	0.005
Post-Ks (5-year)	44.61 (1.62)	45.14 (1.62)	44.12 (1.46)	<0.001
Pre-AL	27.19 (1.47)	26.14 (1.00)	28.15 (1.15)	<0.001
Post-AL (5-year)	27.57 (1.55)	26.49 (1.06)	28.48 (1.30)	<0.001
**△** *Kf*	−0.05 (0.67)	0.05 (0.56)	−0.14 (0.76)	0.120
**△** *Ks*	0.03 (0.66)	0.13 (0.69)	−0.06 (0.62)	0.114
**△**AL	0.28 (0.44)	0.11 (0.39)	0.42 (0.42)	0.001

Ks: keratometry steep; *Kf*: keratometry flat; AL: axial length; △: Post-Pre.

**Table 5.** The correlation between AL increase and preoperative parameters.

Preoperative parameters	PostAL-PreAL	R	*p* Value
Age	△AL	−0.0883	0.427
Kf	△AL	−0.191	0.083
Ks	△AL	−0.109	0.322
SE	△AL	−0.451	0.000
CCT	△AL	−0.129	0.243
WTW	△AL	0.132	0.234
PD	△AL	0.037	0.744
ACD	△AL	−0.069	0.535
IOP	△AL	0.070	0.527
ICL size	△AL	0.195	0.078

Ks: keratometry steep; Kf: keratometry flat; SE: spherical equivalent; CCT: central corneal thickness; WTW: white-to-white distance; PD: pupil diameter; ACD: and anterior chamber depth; IOP: intraocular pressure; AL: axial length; △:Post-Pre.

### 3.5. IOP, ECD, QIRC and vault

Preoperatively, we observed that the average IOP was 13.97 ± 3.09 mmHg in high myopia group. Postoperatively, the mean IOPs were 15.00, 13.21, 14.77, 14.66, 15.38 and 15.19 mmHg at the visit of 1 week, 1 month, 3 months, 6 months, 1 year, and 5 years, respectively. The mean follow-up IOPs in super-high myopia were 16.15, 17.83, 15.61, 16.93, 17.58, 16.80, and 16.69 mmHg at the visit of 1 week, 1 month, 3 months, 6 months, 1 year, and 5 years, respectively (Table 5). Notably, the comparative results of intraocular pressure before and after the surgery have statistical significance, but the difference values are within the physiological fluctuation range of intraocular pressure.

The average ECD was 2848 ± 254 cells/mm^2^ at baseline. Postoperatively, the mean ECDs were 2785 ± 321 and 2841 ± 300 cells/mm^2^ at the visit of 1-month and 5-year, respectively (Table 6). Compared to high myopia group, no significant difference of ECD was found in the super-high myopia.

**Table 6.** Various parameters at different follow-up times after ICL-V4c implantation.

	ALL	High	Super high	
	*N = 125*	*N = 60*	*N = 65*	*p*
QIRC	45.81 (7.41)	46.74 (5.28)	44.93 (8.95)	0.327
ECD (cells/mm^2^)				
Baseline	2847.70 (254.13)	2828.57 (272.58)	2864.77 (237.29)	0.436
1 month	2785.32 (321.72)	2802.73 (301.60)	2769.81 (340.65)	0.602
5 years	2840.74 (300.36)	2823.45 (274.75)	2856.71 (323.53)	0.536
IOP(mmHg)				
Baseline	15.11 (3.45)	13.97 (3.09)	16.15 (3.45)	<0.001
1 week	16.52 (4.32)	15.00 (3.56)	17.83 (4.51)	<0.001
1 month	14.50 (3.43)	13.21 (2.55)	15.61 (3.71)	<0.001
3 months	16.20 (3.46)	14.77 (3.29)	16.93 (3.37)	0.058
6 months	16.12 (3.25)	14.66 (2.32)	17.58 (3.43)	0.004
1 year	16.20 (3.12)	15.38 (3.17)	16.80 (3.00)	0.149
5 years	15.97 (2.80)	15.19 (2.40)	16.69 (2.96)	0.002
Vault(µm)				
1 week	373.5 (126.8)	277.12 (180.59)	312.22 (224.48)	0.342
1 months	373.4 (149.7)	335.66 (131.92)	395.00 (173.62)	0.042
3 months	402.24 (136.32)	388.89 (174.52)	410.00 (110.94)	0.648
6 months	392.31 (162.44)	377.50 (180.26)	407.89 (144.59)	0.564
1 year	379.73 (146.26)	370.71 (137.53)	385.22 (154.09)	0.768
5 years	311.49 (176.58)	262.22 (148.06)	356.97 (189.29)	0.002

QIRC: refractive surgery impact on quality of life questionnaire; ECD: endothelial cell density; IOP: intraocular pressure.

The mean (±*SD*) QIRC was 45.81 (7.41), 46.74 (5.28), and 44.93 (8.95) in all eyes, high and super-high myopic eyes (Table 6). No significant difference of QIRC was found between high and super-high myopia.

We observed that the average vault of all eyes at 1 weeks, 1 month, 3 months, 6 months, 1 year, and 5 years follow-up were 373.5 ± 126.8, 373.4 ± 149.7, 402.2 ± 136.3, 392.3 ± 162.4, 379.7 ± 146.3 and 311.5 ± 176.6 μm, respectively. Interestingly, during postoperative one year, no significant variations were observed in vault. However, the vault size at the visit of 5-year was significantly lower than that at the 1-year follow-up (Table 6). There was a significant difference in vault between the high and super-high groups at 1-month and 5-year after surgery (Table 6).

### 3.6. Complications

Among the 125 follow-up eyes, only one eye presented with cataract opacity. The patient was over 45 years old and had previously undergone PRK surgery for myopia correction over 20 years ago and subsequently underwent ICL implantation surgery due to the recurrence of myopia. The UDVA of this eye was still 0 (logMAR). Two eyes came from the same patient experienced no improvement in CDVA, and they were found to have maculopathy that affected their visual acuity *via* examination of OCT (Supplemental Figure S1). In addition, four eyes exhibited increased intraocular pressure postoperatively at 1 week, with the highest value not exceeding 32 mmHg. After treatment or observation, they improved. Throughout the follow-up period, no serious complications occurred in any of the eyes.

## 4. Discussion

As the incidence of myopia continues to rise in China, more and more patients with refractive errors are choosing refractive surgery for correction. Due to limited corneal conditions, most high myopic patients choose ICL surgery. Compared with the implantation of anterior chamber artificial lenses, ICL implantation has fewer complications and less damage to the cornea and lens, making it an effective and safe method for correcting high myopia [23,25,26]. The ICL-V4c used in this study is a central aperture type that comes with an aqueous humor circulation system and does not require iridotomy before surgery, reducing eye damage in patients with refractive errors and preventing the development of iris obstruction glaucoma [27–29]. During ICL-V4c implantation, a series of operations on the anterior segment of the eye may potentially damage the corneal endothelial cells, so the postoperative status of the corneal endothelial cells needs to be observed to avoid irreversible damage. Since the ICL-V4c lens was only approved by China’s State Food and Drug Administration in 2014 and entered the Chinese market in 2015, there is limited verification of its long-term efficacy after surgery. Therefore, in the 5-year follow-up study, we observed that ICL-V4c implantation is a safe and effective treatment for high and super-high myopia.

This study retrospectively reviewed 64 patients (a total of 125 eyes) who had undergone ICL-V4c implantation for 5 years. After 5 years of surgery, 51 eyes (40.80%) of UDVA (logMAR) were 0.0 or above, 86 eyes (68.88%) were 0.1 or above, and 119 eyes (95.20%) were 0.3 or above. In the analysis of the effectiveness index, the effectiveness index was above 1.0 at 1 week, 1 month, 3 months, 6 months, and 1 year after surgery. At 5 years after surgery, the effectiveness index for high myopic patients was 0.91 ± 0.23, while the effectiveness index for super-high myopic patients was 0.80 ± 0.25, which is similar to the results reported by Chen et al. [24]. who observed EVO implantation for 5 years after surgery. The decrease in effectiveness index was more significant in the super-high myopic group than in the high myopic group over time, mainly due to the increase in axial length. The preoperative axial lengths of the high myopic and super-high myopic groups were 26.14 ± 1.00 mm and 28.15 ± 1.15 mm, respectively. Five years after surgery, the axial lengths of the high myopic and super-high myopic groups were 26.49 ± 1.06 mm and 28.48 ± 1.30 mm, respectively, representing an increase in length of 0.11 ± 0.39 mm and 0.42 ± 0.42 mm. The comparison of the two groups showed statistical significance, which indicated that the visual acuity regression in super-high myopic patients may be greater. This is consistent with the results of Gab-Alla et al.’s analysis of reasons for myopic regression after LASIK, where 86.6% of the cases involved axial lengthening in moderate to high myopic patients [30]. The super high myopia patients with longer axial length tend to experience an increase in axial length [31]. The elongation of the AL is often accompanied by thinning of the sclera, which can contribute to the structural changes observed in super high myopia. Therefore, it can be seen that the higher degree of preoperative super-high myopia, the poorer stability of postoperative visual acuity, and the higher the likelihood of axial elongation and myopia progression.

The safety-related analysis suggests that the postoperative UCVA and CDVA were significantly better than preoperative levels. In some patients, postoperative UCVA is even better than preoperative CDVA, which is similar to the results found by Du et al. [32]. Patients with high myopia who wear frame glasses have thicker lenses and are farther away from the cornea. When light enters the eye, it must first pass through the thick lens to refract, resulting in reduced image quality and unsatisfactory visual effects [33]. However, ICL is implanted in the posterior chamber of the eye, with no distance consumption of light and providing a certain magnification effect on the object itself, resulting in increased resolution of images, and significant improvements in postoperative vision and visual effects [23,34]. Additionally, we observed that only two cases had a decrease in CDVA after surgery, which was found to be associated with maculopathy *via* examination. Furthermore, the 5 years of safety index was 1.10 ± 0.15 for the high myopic group and 1.10 ± 0.21 for the super-high myopic group, indicating that ICL-V4c implantation is safe and effective for the treatment of high and super-high myopic eyes.

The current study revealed that there was no immediate increase in intraocular pressure (IOP) in high and super-high myopic patients who underwent ICL-V4c implantation during the early postoperative period. However, four patients experienced increased IOP at one-week post-surgery, which may have been due to residual viscoelastic agent from intraoperative use, the inflammatory response following intraocular surgery, or the use of steroid eye drops after surgery. It has been previously found that glucocorticoid eye drops can increase IOP, especially in patients with high myopia [35–37]. Consistent with prior research, we attributed the transient increase in IOP observed at 1 week to the use of glucocorticoid eye drops [38,39]. This temporarily elevated IOP subsided after discontinuing medication or through the prescription of eye drops to lower eye pressure. Subsequently, there was a slight decrease in IOP at one month, and no significant difference was observed in IOP at different time points after one month. These findings suggest that ICL-V4c can provide long-term IOP stabilization without requiring previous iridotomy. More interestingly, we observed that IOP was higher in the super high myopia group than in the high myopia group (Table 6). One possible explanation for this finding is that the thicker CCT in the super high myopia group may be a compensatory mechanism for the increased axial length and scleral curvature often associated with high myopia (Table 1). Another possibility is that the super high myopia group may have a higher prevalence of underlying glaucomatous changes, such as increased resistance to aqueous humor outflow or decreased production of aqueous humor [40]. These changes can also contribute to elevated IOP.

In endothelial cell density (ECD), we found that there was no significant difference at various time points after surgery. Previous research has shown a wide range in the rate of endothelial cell loss, ranging from 2.5% to 9.9% across several studies [41–43]. However, this current study suggests that the presence of a central hole does not lead to increased long-term endothelial cell loss in high and super-high myopic patients, and the density of endothelial cells remains within a safe range over the long term.

Having an adequate post-operative vault is crucial, as it ensures there is ample space between the anterior and posterior surfaces of the inserted ICL. Regardless of vault in high and super-high myopic group, we observed that there is a noticeable increase in the vault value at 1 month after surgery, followed by a gradual decline at 3 months after surgery. This phenomenon is speculated to be related to the use of miotic agents during surgery, resulting in a gradual increase in vault in the early stage and followed by a gradual decrease in vault three months later.

Although this study provides valuable insights into the long-term efficacy, safety, and predictability of ICL-V4c implantation in patients with high myopia and super high myopia, there are several limitations that should be acknowledged: 1. Retrospective design: the lack of a prospective design may impact the quality and reliability of the collected data. 2. Limited sample size: the sample size in this study is relatively small, and a larger sample size would enhance the statistical power of the analysis and increase the generalizability of the study results. 3. Single-center study: the study was conducted at a single center, which may limit the generalizability of the study results to other populations or settings.

## 5. Conclusion

In summary, the ICL-V4c is an effective, safe, and stable option for correcting high and super-high myopia. Compared to high myopia, the myopia regression in super-high myopic patients may be greater. The increase in axial length and associated fundus complications still need our attention.

## Supplementary Material

Figure S1.jpg

## Data Availability

The datasets used in the current study are available from the corresponding author on reasonable request.
