# Supplementary figures and images for: Five years comparation of efficacy and safety after ICL-V4c implantation for high and super high myopia correction
Source: Ann Med. 2024 Dec 30;57(1):2448282. doi: 10.1080/07853890.2024.2448282 (PMC11703061; doi:10.1080/07853890.2024.2448282)

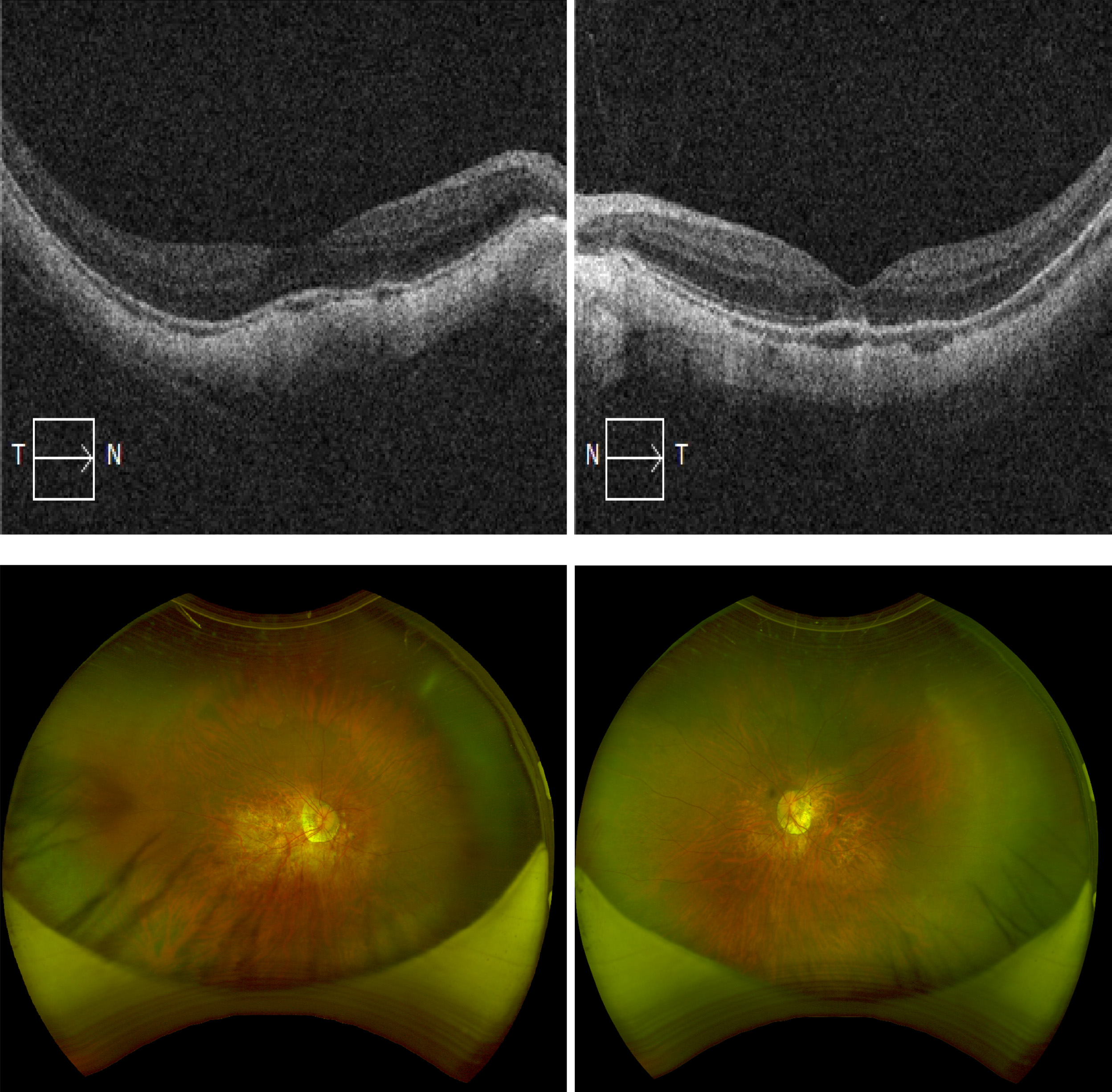

Supplement: Figure S1.jpg [file IANN_A_2448282_SM1430.jpg]
